# Nuclear Motion Is Classical: Spectrum of a Magic Protonated Water Cluster

**DOI:** 10.3390/molecules28186454

**Published:** 2023-09-06

**Authors:** Irmgard Frank

**Affiliations:** Theoretical Chemistry, Leibniz University Hannover, Callinstr. 3A, 30167 Hannover, Germany; irmgard.frank@theochem.uni-hannover.de

**Keywords:** classical nuclear motion, spectra, Car–Parrinello molecular dynamics

## Abstract

The assumption that nuclear motion is classical explains many phenomena. The problems of Schrödinger’s cat and the EPR paradoxon do not exist in a perfectly deterministic theory. All it needs is to describe nuclear motion classically right from the beginning. To establish this simple idea, it must be tested for as many examples as possible. In the present paper, we use ab initio molecular dynamics to investigate the infrared spectrum of a ‘magic’ protonated water cluster H${}_{3}$O${}^{+}$(H${}_{2}$O)${}_{20}$ which exhibits some features that were believed to afford a quantum treatment of nuclear motion. The role of the temperature in contrast to a quantum mechanical description is discussed.

## 1. Introduction

It is perfectly clear that all kind of matter is quantized in the sense that at normal energies everything consists of atoms or, more precisely, of tiny nuclei and an extended electronic wave function. We got used to this picture not only by theoretical considerations, but, thanks to modern microscopy and spectroscopy, also by direct experimental observations of single molecules. Nevertheless, it is not so clear if quantization means that absolutely everything should be described with the Schrödinger equation as proposed by the Copenhagen interpretation. In fact, such a comprehensive use of the Schrödinger equation leads to the well-known paradoxa. These paradoxa are at odds with observation, which is most obvious if one has a look at chemical reactions. We are able to simulate such reactions and to observe how every atom gets from the educt state to the product state without needing any measurement process. The approach we employ is ab initio molecular dynamics (AIMD) using the Car–Parrinello scheme (CPMD) [1,2]. Molecular dynamics (MD) means that we are using Newton dynamics for the nuclei. Ab initio molecular dynamics means that for the description of the electronic cloud the Schrödinger equation is used in a molecular dynamics run. This approach is local and deterministic. Simulating the same reaction two times leads to exactly the same result. Sligthly changing the initial conditions, however, may lead to a completely different product state. We have electronic tunneling and classical chaos for nuclear motion.

The derivation of CPMD can be put in a way as to make no use of the Born–Oppenheimer approximation in order to separate nuclear and electronic motion. The Born–Oppenheimer approximation attributes a wave function to the nuclei and a wave function to the electrons and describes the total wave function as the product of these two wave functions. This would make sense if the problem was separable into an electronic and a nuclear problem. It turns out, it is not. The electronic–nuclear interaction is not negligible and the nuclear–nuclear interaction cannot be omitted if one wants to obtain potential energy surfaces. See, for example, the code in [3].

A more direct derivation starts from assuming the nuclei as classical particles. Note that this does not mean that the nuclei have no structure and no properties at all. An inner structure of the nuclei just does not matter for investigating nuclear motion like it appears in chemical reactions. There is no reason to assume that the nuclear structure obeys the same laws as the electronic structure does. The nuclei are smaller than the lighter electrons, in agreement with the deBroglie relation. However, the heavier the nuclei get in the periodic table, the larger they become while the density stays about the same. A naive application of the deBroglie picture would lead to a clear deviation from the experiment. The nuclei are clearly different from the electrons, also in the sense that they can be discriminated. For the motion in an electrical field generated by the electrons and all other nuclei, we need only their masses and charges.

Matter of any kind consists of atoms, which again consist of electrons and nuclei. The total Hamiltonian for a system with N electrons and M nuclei is:

(1) $${\hat{H}}_{total}={\hat{T}}_{nuc}+{\hat{T}}_{elec}+{\hat{V}}_{nuc-nuc}+{\hat{V}}_{elec-nuc}+{\hat{V}}_{elec-elec}$$

${\hat{T}}_{nuc}$ is the kinetic energy of the nuclei, ${\hat{T}}_{elec}$ is the kinetic energy of the electrons, ${\hat{V}}_{nuc-nuc}$ is the nuclear-nuclear interaction, ${\hat{V}}_{elec-nuc}$ is the electronic–nuclear interaction, and ${\hat{V}}_{elec-elec}$ is the electronic–electronic interaction. A normal quantum mechanical calculation uses, for example, the density functional theory (DFT) approximation in the Kohn–Sham (KS) formalism for the description of the electronic structure. In the following, the terms ‘density functional theory’ and ‘Kohn–Sham’ will be used synonymously. In such a DFT calculation, the kinetic energy of the nuclei is put to zero. Since the kinetic energy of the nuclei corresponds to the temperature of the system, this means modelling the situation at zero Kelvin. We obtain ${\hat{H}}_{QC}$ which contains, besides all electronic terms, the nuclear–nuclear interaction $V_{nuc-nuc}$. This term is missing in the traditional derivation according to Born and Oppenheimer as a consequence of separating electronic and nuclear wavefunctions.

(2) $${\hat{H}}_{QC}={\hat{T}}_{elec}+{\hat{V}}_{nuc-nuc}+{\hat{V}}_{elec-nuc}+{\hat{V}}_{elec-elec}$$

A separation would lead to the electronic Hamiltonian ${\hat{H}}_{el}$.

(3) $${\hat{H}}_{el}={\hat{T}}_{elec}+{\hat{V}}_{elec-nuc}+{\hat{V}}_{elec-elec}$$

${\hat{H}}_{el}$, however, does not describe proper Born–Oppenheimer surfaces, in contrast to ${\hat{H}}_{QC}$. Nevertheless, it is sufficient for deriving equations of motions, because $V_{nuc-nuc}$ yields zero when varying the energy expression with respect to the wavefunction.

The derivation of the Car–Parrinello equations starts with an extended Lagrangian using $E_{QC}$ as obtained from ${\hat{H}}_{QC}$ (capital letters are used for nuclear quantities):

(4) $$L_{CP}=\sum_{I}\frac{1}{2}M_{I}{\underline{\dot{R}}}_{I}^{2}+\sum \mu_{i}\int {\dot{\psi }}_{i}^{*}{\dot{\psi }}_{i}d\tau -E_{QC}+\sum_{i}\sum_{j}\Lambda_{ij}\left(\int \psi_{i}^{*}\psi_{j}d\tau -\delta_{ij}\right)$$

The four components of the Car–Parrinello Lagrangian are as follows: $\sum_{I}\frac{1}{2}M_{I}{\underline{\dot{R}}}_{I}^{2}$: kinetic energy of the classical nuclei, $\sum \mu_{i}\int {\dot{\psi }}_{i}^{*}{\dot{\psi }}_{i}d\tau$: fictitious kinetic energy of the electrons, $E_{QC}$: energy obtained from a quantum chemical calculation of the electronic structure using ${\hat{H}}_{QC}$, $\sum_{i}\sum_{j}\Lambda_{ij}\left(\int \psi_{i}^{*}\psi_{j}d\tau -\delta_{ij}\right)$: constraints introduced to guarantee orbital orthonormality during the simulation.

Hereby is:

(5) $$E_{QC}=E_{KS}+V_{nuc-nuc}$$

$E_{KS}$ is the Kohn–Sham energy computed for the electronic Hamiltonian ${\hat{H}}_{el}$ in density functional approximation, containing the kinetic energy of the electrons, the interaction of the electrons with the nuclei and the electron–electron interaction. $E_{QC}$ is the energy computed in a normal quantum chemical calculation, here: density functional calculation. $E_{QC}$ contains all relevant terms except the kinetic energy of the nuclei. It is obtained without making use of a nuclear wave function. The kinetic energy of the nuclei is added in the Car–Parrinello Lagrangian (first term in the Lagrangian) under the assumption of classical particles. Like this, non-zero-temperature situations are modelled. Note that, by adding this classical version of ${\hat{T}}_{nuc}$, all five terms of the energy expression ${\hat{H}}_{total}$ we started from are considered.

The corresponding Euler–Lagrange equations read:

(6) $$Nuclei:\frac{d}{dt}\frac{\partial L}{\partial {\underline{\dot{R}}}_{I}}=\frac{\partial L}{\partial {\underline{R}}_{I}}$$

(7) $$Electrons:\frac{d}{dt}\frac{\delta L}{\delta {\dot{\psi }}_{i}^{*}}=\frac{\delta L}{\delta \psi_{i}^{*}}$$

This results in the following Car–Parrinello equations of motion:

(8) $$Nuclei:M_{I}{\underline{\ddot{R}}}_{I}=-\frac{\partial }{\partial {\underline{R}}_{I}}E_{QC}$$

(9) $$Electrons:\mu_{i}{\ddot{\psi }}_{i}=-\left[-\frac{1}{2}\nabla^{2}+v_{eff}\left(\underline{r}\right)\right]\psi_{i}+\sum_{j}\Lambda_{ij}\psi_{j}$$

In deriving the latter equation, the Kohn–Sham approximation was used. It can be read in the following way: The deviation from the Kohn–Sham equations as a consequence of nuclear motion (right side) leads to a force on the orbitals (left side of the equation). Note that the Car–Parrinello equations contain second derivatives both for space and time. While the kinetic energy of the orbitals was introduced ad hoc, it comes as no surprise that something was missing in the time-independent description of electronic structure using the time-independent Schrödinger equation. The well-working Car–Parrinello equations may be similar to a more fundamental equation which is not yet known. It is remarkable that the CPMD equations which contain neither imaginary parts nor single derivatives with respect to time can be solved in a straight-forward way and yield a convincing picture.

The alternative to Car–Parrinello molecular dynamics is so-called Born–Oppenheimer dynamics (BOMD). In this variant of ab initio molecular dynamics, no forces on the orbitals are computed. Instead, the electronic wave function is fully optimized to the ’Born–Oppenheimer’ surface in every time step. It is the more widely used variant, because the implementation is much easier than that of the more efficient Car–Parrinello molecular dynamics. The implementation of CPMD is particularly difficult if Gaussian basis sets are used instead of plane-wave basis sets, due to an additional term taking into account the motion of atom-centered basis functions through space during a dynamics run. For the present consideration, it does not matter if CPMD or BOMD is used, because the treatment of nuclear motion is the same in both approaches.

**Figure 1:**
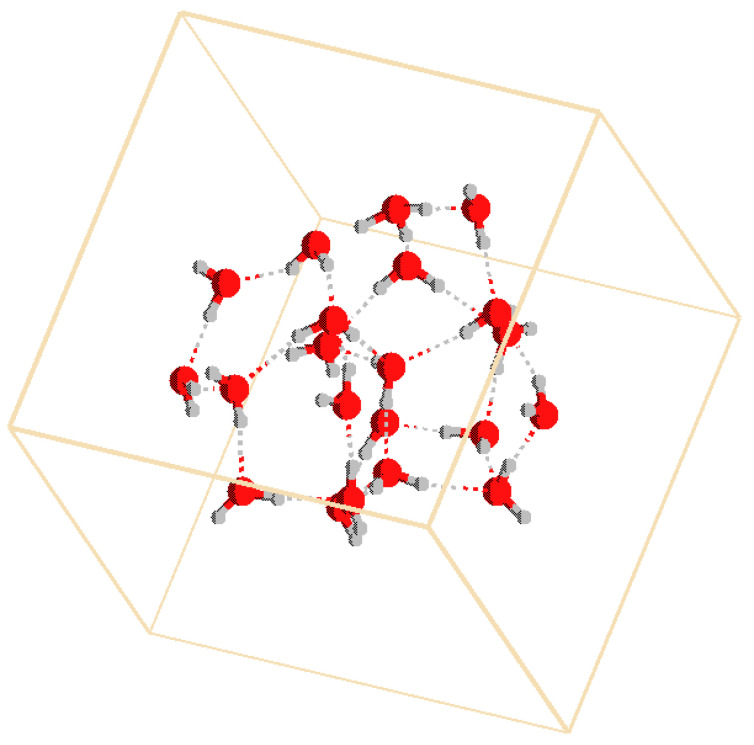
Structure of the water cluster H${}_{3}$O${}^{+}$(H${}_{2}$O)${}_{20}$. The H${}_{3}$O${}^{+}$ is at the top of the cluster. Color code: red: oxygen, grey: hydrogen. The smaller one of the two unit cells is depicted.

With the simple idea that nuclear motion is classical, we have arrived at a local and deterministic theory. While it is easy to formulate this new view of quantum chemistry, it is not so easy to show that it is in agreement with experiment. Many phenomena have been attributed to a quantum nuclear motion. This has a long history [4,5,6], but there are also important developments which have been published recently [7,8,9]. It must be shown one by one that such situations can also be explained by nuclear motion at finite temperatures. See our previous publications [10,11,12,13,14,15]. Infrared (IR) spectra are among the most important phenomena that may be attributed to quantum nuclear motion. In the present study we employ CPMD to compute spectra of a water cluster H${}_{3}$O${}^{+}$(H${}_{2}$O)${}_{20}$ [16,17] that was found to be particularly stable (Figure 1). Its characteristic IR spectrum contains some features that were attributed to quantum motion of the nuclei. We will show that this assumption is not needed and that temperature is a sufficient concept to explain the observations.

## 2. Results and Discussions

We performed a total of 27 CPMD simulation runs (see Table 1 and the Section 4 below).

We find an Eigen structure, which means that we have in essence H${}_{3}$O${}^{+}$ instead of H${}_{5}$O${}_{2}^{+}$. This is in agreement with previous calculations [17]. See also [18]. Zundel structures, H${}_{5}$O${}_{2}^{+}$, are observed as transition states when H${}_{3}$O${}^{+}$ exchanges a proton with a neighbouring water molecule, which happens several times in the simulation runs.

**Figure 2:**
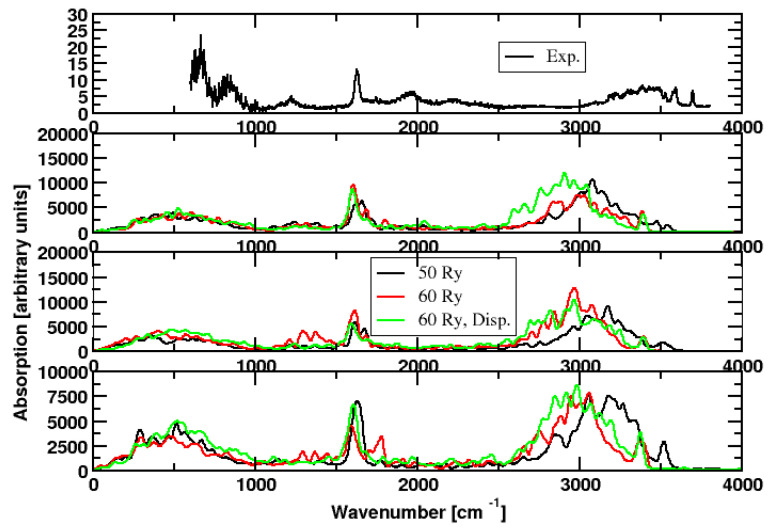
**First panel**: Experimental spectrum. CPMD simulations: BLYP, temperature ions 300 K. Color code: black: plane-wave cutoff 50 Rydberg, red: plane-wave cutoff 60 Rydberg, green: plane-wave cutoff 60 Rydberg, dispersion correction added. **Second panel**: cell size 12.70 × 12.70 × 12.70 Å${}^{3}$). **Third panel**: cell size 15.87 × 15.87 × 15.87 Å${}^{3}$. **Forth panel**: cell size 15.87 × 15.87 × 15.87 Å${}^{3}$, counterion added.

We started with nine simulations that tested the effect of the super-cell size as well as the addition of a chlorine atom as counter ion. (If there is no counter ion, CPMD uses a background charge to describe the periodically repeated charged system). Each of these scenarios was computed for plane-wave cutoffs of 50 and 60 Rydberg. In part of the simulations, a dispersion correction was added. See Table 1. The result is described in Figure 2. Three phenomena are most prominent: the H${}_{2}$O libration below 1000 cm${}^{-1}$, the water peak at about 1600 cm${}^{-1}$, and the OH stretch vibration at 3000 cm${}^{-1}$ and above. All three scenarios (plane-wave cutoff of 50 and 60 Rydberg and dispersion correction), do not change the picture strongly. An exception is the OH stretch vibration. Here, increasing the cutoff and introducing a dispersion correction brings the peaks farther away from experiment. This is attributed to shortcomings of the BLYP functional. It should be emphasized, however, that overall BLYP is doing a great job, particularly if one considers the cost/value ratio.

The super cell size and the addition of a counter ion have little effect. In other words, the cell is big enough and the background charge applied to the charged system does not influence the result.

Most important is the comparison of the weak H${}_{3}$O${}^{+}$ peaks to experiment, because these peaks are believed to be influenced by quantum mechanical nuclear motion [17]. With our classical approach, we observe weak features close to 1200 cm${}^{-1}$ and around 2000 cm${}^{-1}$. However, this observation is a bit accidental. In some of the simulations, some of these peaks are missing. There is no clear trend, hence we assume that this is due to the accidental observation of a particular vibration on the picosecond timescale. It is simply unlikely, that all relevant vibrations are excited during the short simulation time span in a single cluster.

**Figure 3:**
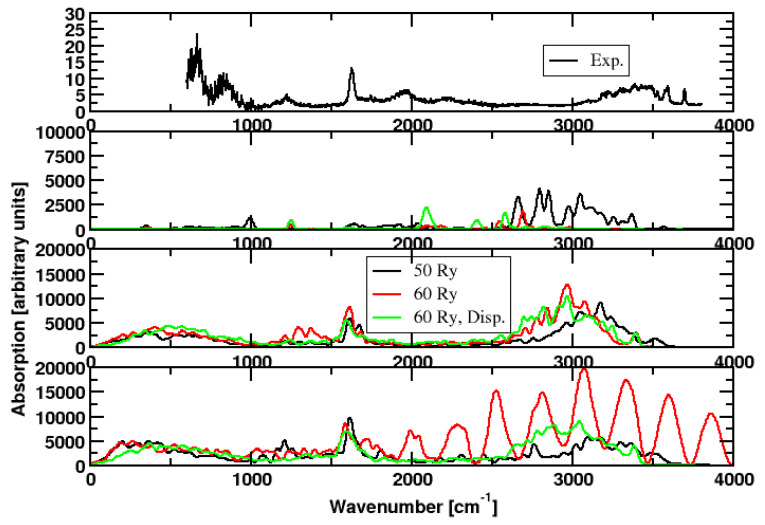
**First panel**: Experimental spectrum. CPMD simulations: BLYP, cell size 15.87 × 15.87 × 15.87 Å${}^{3}$. Color code: black: plane-wave cutoff 50 Rydberg, red: Plane-wave cutoff 60 Rydberg. Green: plane-wave cutoff 60 Rydberg, dispersion correction added. **Second panel**: start temperature of the ions is 0 K. **Third panel**: start temperature of the ions is 200 K. **Forth panel**: start temperature of the ions is 400 K. The outlier (red curve in the forth panel) is due to a decomposition of the cluster at high temperatures.

In a second suite of nine simulations, we tested the effect of temperature (Table 1 and Figure 3).

Additionally, here, most spectra are similar, whereby the spectra computed close to 0 K show the largest deviations. In these simulations, the water peak does not appear at all. In contrast, the weak features close to 1200 cm${}^{-1}$ and 2000 cm${}^{-1}$ are obtained correctly. A further exception represents one of the runs at 400 K (simulation **17**): At this high temperature, the water cluster can decompose on a picosecond timescale, resulting in a clearly different spectrum with the O-H stretch vibration becoming most important.

**Figure 4:**
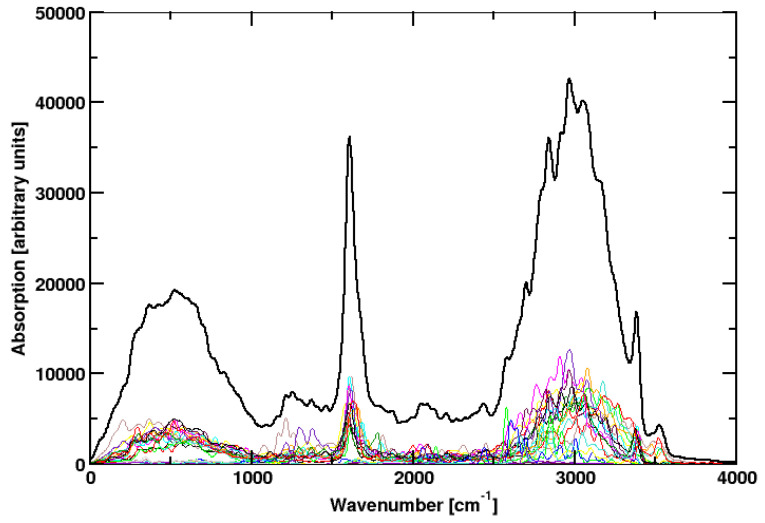
Colored lines: IR spectra of simulation runs **1** to **18** The outlier **17**, which was observed at high temperatures, was omitted. Black line: sum over all these spectra.

To summarize, the change of all these parameters affected the results relatively little. Sometimes a peak is missing. Obviously not all vibrational excitations can be observed for a single cluster in the gas phase on the picosecond timescale. Consequently, we put all simulations together in a single graph (Figure 4), omitting the outlier at 400 K. A similar procedure was applied before to compute the spectra of HCl and NH${}_{3}$ [15]. We get a spectrum that compares very well to the experimental spectrum. All relevant features are observed. This procedure has some justification by the fact that we simulate isolated water clusters on the picosecond timescale. Without energy exchange with other clusters, even a way larger amount of CPU time would not lead to complete spectra. We can only do simulations with different initial conditions and simulation parameters.

**Figure 5:**
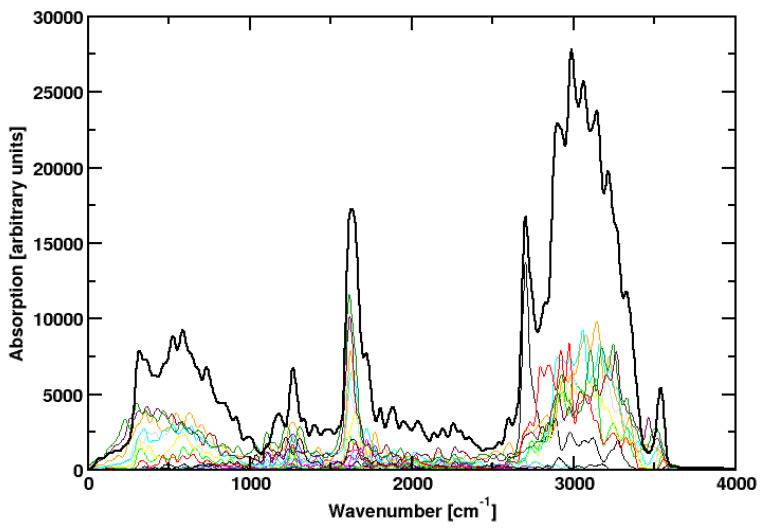
Colored lines: IR spectra of all simulation **19** to **27**. Black line: sum over all these spectra.

To check if this procedure is reproducible, a third suite of simulations was run (Table 1). This time only the temperature was varied, all other parameters stayed the same. The result (Figure 5) is in beautiful agreement with experiment. In particular, the weak features around 1200 cm${}^{-1}$ and around 2000 cm${}^{-1}$ are now perfectly reproduced.

## 3. Discussion

AIMD simulations are able to describe IR spectra [19,20,21,22]. There is no need for a quantum mechanical approach. Of course, the electrons must be described using the Schrödinger equation, but there is no reason to do that also for the nuclei. In contrast, applying the Schrödinger equation to the nuclei leads to paradoxa. One of the most obvious examples that there is something wrong with the Copenhagen interpretation is actually the structure of ammonia. The ammonia inversion is described by a double-well potential. If the nuclei had a wavefunction in the sense of Schrödinger, both minima were equally occupied in the ground state, leading to a D${}_{3h}$ structure, while the experiment clearly proves a C${}_{3v}$ structure with a permanent dipole moment.

With the CPMD code we have a powerful tool in our hands, which, however, is not perfect. Deviations from the experiment are mainly attributed to shortcomings in the description of the electronic many-bodied problem, not in the classical description of nuclear motion. In the present example, the O-H stretch vibrations are shifted to lower wave numbers. This can be attributed to the use of the BLYP functional. BLYP, as a GGA (generalized gradient approximation) functional, has a very good cost-value ratio, but clearly does not have the accuracy of a hybrid functional. We are limited to the use of GGA functionals in our simulations because we use large plane-wave basis sets which makes the use of exact exchange problematic. Nevertheless, the BLYP error is preferable to the problems one encounters when moving atom-centered basis functions through space during dynamics.

In some graphs, not all features of the IR spectrum appear. We clearly have a problem with ergodicity stemming from limited CPU time. Nevertheless, from summing all simulations, it can be seen that the classical description of nuclear motion is in good agreement with the experimental spectrum. There is no peak that could not be explained with ab initio molecular dynamics. In view of practical calculations, however, it has some justification to use a quantum mechanical description of the vibrational wave function and to obtain a complete spectrum in a single calculation, instead of doing many similar CPMD simulations at non-zero temperatures. Additionally, of course, for small systems it is appealing not to be restricted to the accuracy of GGA functionals.

This observation does not alter the statement that nuclear motion is classical. At normal energies, i.e., as long as relativistic effects are not to be expected, Newton’s equations of motion are sufficient. An extension in the sense of special relativity is straight-forward, but there we get into a regime which is not covered by the daily experience of a chemist. Hence, we ignore relativity for the moment. This concerns of course the motion of the nuclei, not the electronic structure, where relativistic effects may be important.

What does a classical motion of the nuclei mean in practice? Our theory is local and deterministic. On this basis, some quantum mechanical paradoxa can be explained. Consider the thought experiment of separating a diatomic such as molecular hydrogen or hydrogen fluoride by irradiation with light. The products are separated from each other in closed boxes. In our local and deterministic theory, it is perfectly clear, at every point in time, which atom is on which side. This does not depend on the question, if the diatomic consists of like atoms such as in molecular hydrogen or of different atoms such as in hydrogen fluoride. The centers of these atoms move deterministicly, independent of a particular experiment. The electronic wavefunction surrounding these centers forms one big wavefunction with all the electrons of the boxes containing the atoms. It is not possible to define the box as something that would not let information through. It may be possible in a mathematical construct, to imagine that the inner of the box is not part of our universal wavefunction. In any physical experiment, the box and its contents are clearly part of our physical universe at every point in time. Hence, it is always clear, which atom is where.

Likewise, every atom of Schrödinger’s cat moves deterministicly at any point in time. From the heat flux through the box it is always clear, if it is dead or alive.

What about the double slit experiment? Feynman’s thought experiment [23] is particularly difficult to understand even if one is open to understanding the Copenhagen interpretation. Actually, it turns out that what Feynman describes is difficult to realize in an experiment, even if it is meanwhile possible to observe and manipulate single molecules. A pure double slit for atoms or molecules has never been constructed; the deBroglie wavelength is simply too small. Zeilinger’s experiments on fullerenes deal with a 100 nm grating, not with a double slit [24]. Fullerenes have a size in the order of 1 nm and this cannot be changed by any experiment, unless the molecules are destroyed. Fullerenes have certainly quantum properties, in the sense that one cannot cut them into halves without strongly changing their properties. In addition, they certainly have an electronic wavefunction surrounding them. However, if they are shot against a double slit, every atom will pass either the one slit **or** the other—unless the slits are smaller than 1 nm, then the fullerenes will hardly reach the other side. So, while Zeilinger’s experiment is interesting, it must be clearly stated, that the scenario is not (and is not intended to be) a confirmation of Feynman’s double-slit thought experiment.

What about the double-slit experiment for electrons? There is surprisingly little known in the literature, while lots of double-slit experiments have been performed for light. What would the thought experiment look like in a modern many-particle view? Electrons moving through a double slit become, at least for a short time, part of the electronic wave function of the double slit or rather of the material surrounding the double slit. It makes little sense to ask if they went through the one **or** the other slit. In a perfectly symmetric scenario, they may move through both slits with equal amounts at the same time. However, that is not the full story: depending on the electron energy, on the double-slit material, and on the double-slit geometry, a larger amount of electron density will pass through the surrounding material. Depending on the conditions, the electron may also be swallowed completely by the sea of electrons forming the double slit, which for simplicity is assumed to be grounded.

To conclude, the notion that nuclear motion is classical while the electronic cloud must be described with the time-independent Schrödinger equation, leads to a simple and compelling model of matter at normal energies. For other situations, the Maxwell equations may represent an important alternative. We still do not have the one single equation that describes everything. In any case, further calculations are necessary to show where the Schrödinger equation must be used for describing nuclear motion and where classical motion at finite temperatures is an alternative concept. Till now, we always found an alternative explanation, see, for example, [13].

## 4. Materials and Methods

Car–Parrinello molecular dynamics simulations using the CPMD code [1,25,26] have been performed in the NVE ensemble using the Becke–Lee–Yang–Parr (BLYP) functional [27,28]. In part of the simulations, the Grimme dispersion correction was applied [29]. The time step was chosen as 1 a.u. (0.0242 fs) and the fictitious electron mass as 100 a.u. These low values were used in order to guarantee an accurate motion of the light hydrogen atoms. Troullier–Martins pseudopotentials as optimized for the BLYP functional were employed for describing the core electrons [30,31]. The plane-wave cutoff which determines the size of the basis set, was set to 50.0 or 60.0 Rydberg. The simulation cell size was 24 × 24 × 24 a.u.${}^{3}$ (12.70 × 12.70 × 12.70 Å${}^{3}$) or 30 × 30 × 30 a.u.${}^{3}$ (15.87 × 15.87 × 15.87 Å${}^{3}$). Additionally, these values are relatively large for the small systems investigated. The aim is not to lose accuracy through limited basis sets and cell sizes. After geometry optimization, simulation runs were performed with initial kinetic energies of 300 K (simulations **1** to **9**, see Table 1). Trajectories were determined for 1,000,000 steps per simulation run, that is, for 24 ps. In a second suite of simulations, the temperature was varied between 0 and 400 K, while in part of the calculations, the pseudopotential cutoff was changed and the Grimme correction was applied (simulations **10** to **18**). The last suite of simulations (simulations **19** to **27**) was performed as a check for temperatures varying between 0 and 400 K, while all other parameters remained unchanged. The TRAVIS program [22,32] was used to compute the spectra from the trajectories. The TRAVIS code computes the spectra from the autocorrelation of the dipole moments [21,33]. Both the contributions of nuclei and orbitals to the dipole moment are taken into account. The orbital contribution is included via localized Wannier orbitals, as obtained from AIMD simulations [34,35,36]. For the computation of the dipole moments, only the centers of these localized orbitals are needed.

Sample input files are provided for CPMD and for TRAVIS in the Supplementary Material. Sample job files for running CPMD in a parallel environment are also added.

Table 1 lists the parameters used in the single simulation runs.

## 5. Conclusions

If nuclear motion is classical, it must be possible to describe infrared spectra on the basis of ab initio molecular dynamics simulations. The present investigation of H${}_{3}$O${}^{+}$(H${}_{2}$O)${}_{20}$ shows once more that ab initio molecular dynamics simulations present a reliable basis for the determination of infrared spectra. This confirms previous results [15]. It is important to state that a single simulation at the pico-second time scale is hardly sufficient to simulate a spectrum. Instead the results of several simulation runs with different starting conditions must be added together. This was found for gas-phase systems with relatively few degrees of freedom. For large systems in the condensed phase, a single simulation may be enough. However, it is also recommended in this case to test the effect of temperature. The accuracy of our calculations is limited by the use of density functionals in the GGA approximation. Nevertheless, for infrared spectra of large and complex systems, ab initio molecular dynamics simulations using GGA functionals can even be the best choice.

## Figures and Tables

**Table 1:** Parameters in the single simulation runs.

Number of Simulation Run	Cell Size	Temperature	Plane Wave Cutoff	Dispersion Correction	Counter Ion
	[Ångstrom]	[Kelvin]	[Rydberg]		
1	12.70	300	50	no	no
2	12.70	300	60	no	no
3	12.70	300	60	yes	no
4	15.87	300	50	no	no
5	15.87	300	60	no	no
6	15.87	300	60	yes	no
7	15.87	300	50	no	yes
8	15.87	300	60	no	yes
9	15.87	300	60	yes	yes
10	15.87	0	50	no	no
11	15.87	0	60	no	no
12	15.87	0	60	yes	no
13	15.87	200	50	no	no
14	15.87	200	60	no	no
15	15.87	200	60	yes	no
16	15.87	400	50	no	no
17	15.87	400	60	no	no
18	15.87	400	60	yes	no
19	12.70	0	50	no	no
20	12.70	50	50	no	no
21	12.70	100	50	no	no
22	12.70	150	50	no	no
23	12.70	200	50	no	no
24	12.70	250	50	no	no
25	12.70	300	50	no	no
26	12.70	350	50	no	no
27	12.70	400	50	no	no

## Data Availability

More input files for CPMD version 4.3 are available on demand from the corresponding author.

## Supplementary Materials

The following supporting information can be downloaded at: https://www.mdpi.com/article/10.3390/molecules28186454/s1, sample CPMD version 4.3 input files in1 (equilibration) and in2 (production), sample job files job1 and job2, sample TRAVIS input file input.txt.

## Abbreviations

The following abbreviations are used in this manuscript:

AIMD	Ab Initio Molecular Dynamics
BLYP	Becke–Lee–Yang–Parr density functional
BOMD	Born–Oppenheimer Molecular Dynamics
CPMD	Car–Parrinello Molecular Dynamics
DFT	Density Functional Theory
GGA	Generalized Gradient Approximation
KS	Kohn–Sham
